# Workplace gaslighting: a construct for organizational research

**DOI:** 10.3389/fpsyg.2026.1589063

**Published:** 2026-03-23

**Authors:** Neha Popat, Jatin Pandey

**Affiliations:** Indian Institute of Management Indore, Indore, India

**Keywords:** affliction, deviant behavior, mistreatment, self-doubt, trivialization, workplace gaslighting, being gaslit

## Abstract

Scholarly interest in gaslighting research has surged across various domains, particularly after it was named the word of the year in 2022. Despite growing research on the topic of gaslighting, the literature on workplace gaslighting remains limited, hindering the establishment of a clear, definitive concept. We review current scholarship on gaslighting through a systematic literature review and analyze definitions across various disciplines. The thematic analysis of definitions was used to delineate the attributes of the gaslighting phenomenon into necessary and contingent. We conceptualize that workplace gaslighting phenomenon comprises of necessary gaslighting behaviors by gaslighters and contingent experience of gaslighting by gaslightees. Gaslighting behaviors have necessary attributes of: (a) trivialization, i.e., minimizing employees’ work and non-work-related concerns and (b) affliction potential, i.e., ability to cause pain, distress, suffering or negative outcome. The experience of gaslighting can escalate to distortion of reality/confusion/self-doubt which is contingent upon perceived power imbalance. Due to the pervasive and covert nature of the construct proving of intention is not always possible, hence intentionality is considered contingent. Based on our findings, we propose a definition of workplace gaslighting and suggest areas for future research.

## Introduction

The financial cost of workplace mistreatment has been estimated to be approximately between “$691.70 billion to $1.97 trillion per annum” (Dhanani et al., 2021, p. 1082). Bowling and Beehr (2006) used the term workplace mistreatment to refer to “interpersonal behavior aimed at intentionally harming another employee in the workplace” (Bowling and Beehr, 2006, p. 998). In a recent meta-analysis, workplace mistreatment was construed to include multiple negative behaviors, namely, “abusive supervision, bullying, discrimination, exclusion/ostracism, general harassment, incivility, interpersonal conflict, psychological aggression, sexual harassment, undermining, and violence” (Dhanani et al., 2021, p. 1083) and it was found that on an average, 34% of employees reported experiencing mistreatment. However, another form of latent workplace mistreatment exists in the form of workplace gaslighting, which is covert in its manifestation and becomes apparent only through interpretation and evidentiary accumulation. While overt workplace mistreatment can be easy to identify, subtle forms of mistreatment such as workplace gaslighting are often difficult to recognize making it challenging to prevent and address. Developing a nuanced understanding of the underlying dynamics of gaslighting is therefore critical for organizations seeking to effectively recognize and address it.

Gaslighting has gained such popularity in media that the Merriam-Webster dictionary named it the word of the year for 2022 due to users’ high level of interest throughout the year. The term “gaslighting” originated in the 1930s and gained recognition with Patrick Hamilton’s play of the same name, which was adapted into two other films (Abramson, 2014). Initially, scholars focused on the occurrence of gaslighting within intimate relationships (Gass and Nichols, 1988; Sweet, 2019). In recent years, there has been a surge of interest in studying gaslighting in the contexts of politics (Abu-Laban and Bakan, 2022; Beerbohm and Davis, 2023), healthcare (Sebring, 2021; Walkden, 2023; Watson-Creed, 2022), psychology (Khan et al., 2024; Tormoen, 2019), philosophy (Abramson, 2014; Catapang Podosky, 2021) and sociology (Bates, 2020; Sweet, 2019) as evidenced by its inclusion in academic journals. In the workplace domain, a scale for measuring gaslighting at work was only developed recently (Kukreja and Pandey, 2023) which was later translated into Persian (Gheshlagh et al., 2025) and Turkish (Güleç and Özbay, 2024) versions. Gaslighting at work has been found to have adverse outcomes such as negative correlation with career entrenchment of nurses (Atta et al., 2025), and decreased life satisfaction in Turkish women (Güleç and Özbay, 2024). In another qualitative study, racial gaslighting in academia was found to disproportionately silence women of color through invalidation and denial (Patterson and Matias, 2025).

Despite a surge in research related to gaslighting, the subject is under-researched and under-theorized (Jones, 2023; Omran and Yousafzai, 2023) especially in the workplace domain. Even though the scale of gaslighting at work has been developed, there is no definition and conceptual clarity around the phenomenon. Podsakoff et al. (2016) highlight the necessity of a definition to foster scientific advancement but there still remains a lacuna in the conceptualization of workplace gaslighting.

In this paper we undertake a literature review to understand different conceptualizations of gaslighting. Based on definitions from scholarly discourse, we conceptualize workplace gaslighting phenomenon as a combination of gaslighting behaviors by the perpetrator (actions) and experience of gaslighting by the target (perceptions). We distinguish gaslighting from related constructs such as workplace bullying, incivility, abusive supervision, and social undermining. Through this paper, we aim to advance research on workplace gaslighting by clarifying and delineating the concept.

Our paper makes following contributions. We delineate gaslighting phenomenon into its components, i.e., the gaslighting behaviors (action) and experience of gaslighting (perception). By dividing the gaslighting phenomenon into action and perception, we highlight that gaslighting tactics used in the workplace comprise the gaslighting behavior irrespective of the impact (perception) on the gaslightee which is contingent on power imbalance. We posit that similar actions can differently impact individuals based on perceived power imbalance (Abramson, 2014; Dorri et al., 2024; Li and Samp, 2023; Sweet, 2019; Wood and Harris, 2024). This paper aids in identifying gaslighting behaviors in the workplace, which may not be readily apparent due to their manipulative nature (Sweet, 2019). Our paper adds to the growing literature on workplace mistreatment (Dhanani et al., 2021; Dhanani and Bogart, 2025).

We structure the paper into stages of definition development. The first stage involves conducting a systematic literature review to compile a comprehensive repository of gaslighting definitions from scholarly research. This is followed by identifying potential attributes associated with gaslighting. Next, we categorize these attributes by theme to distinguish necessary attributes from those that may be present but are contingent. Next, we define workplace gaslighting and differentiate it from other forms of workplace mistreatment. We discuss the existing theoretical perspectives on gaslighting. We conclude the paper with a discussion, recommendations and future research directions.

## Stage 1: systematic literature review

In this phase, we undertake a systematic literature review (SLR) of the papers published on gaslighting to create a repository of conceptualizations of gaslighting. Management has gradually adopted SLRs to promote clarity, transparency, and unbiased coverage of specific areas (Khan and Pandey, 2023; Thorpe et al., 2005). SLR distinguishes itself from traditional literature reviews by emphasizing objectivity, replicability, comprehensiveness, and a systematic approach that mirrors empirical research. SLR compiles and synthesizes existing research, offering a comprehensive and current synthesis of the prevailing knowledge in the field (Palmatier et al., 2018). In this study, the SLR focuses on research that has explored gaslighting. Unlike domain-specific SLR, which limits the review to only papers from a specific domain, we have reviewed papers from various domains since gaslighting is still an evolving and under-researched construct (Omran and Yousafzai, 2023).

A search was conducted across prominent academic databases such as Web of Science and Scopus to ensure comprehensive coverage (He et al., 2024; Linnenluecke et al., 2020). Web of Science and Scopus are recognized for their comprehensive repository of peer-reviewed scholarly articles (Li et al., 2010).

### Inclusion and exclusion criteria

The following inclusion/exclusion criteria were used in this analysis (Okwir et al., 2018):

- articles published in journals listed on the Australian Business Deans Council (ABDC) list (Bapuji et al., 2024), Academic Journal Guide (AJG) list (Koburtay et al., 2019), and Q1 ratings as per Scientific Journal Rankings (SJR) (Schätzlein et al., 2023) (in the areas of Business, Management & Accounting, Arts and Humanities, Psychology, Decision Sciences, Multidisciplinary, social sciences);
- articles available up to 31 December 2024; and
- relevance to the research topic.

### Search methods

The term “gaslight*” was used as a search keyword across the databases searched across all sections of the articles. The search yielded 297 results from WOS and 1,864 from Scopus (i.e., a total of 2,161). We excluded books, book reviews, and editorials since these are generally not subject to peer review. Articles published in journals listed on the ABDC, AJG, or ranked Q1 in SJR were retained. Following this curation and removal of duplicates, 884 articles emerged and were subjected to manual scrutiny.

**Figure 1:**
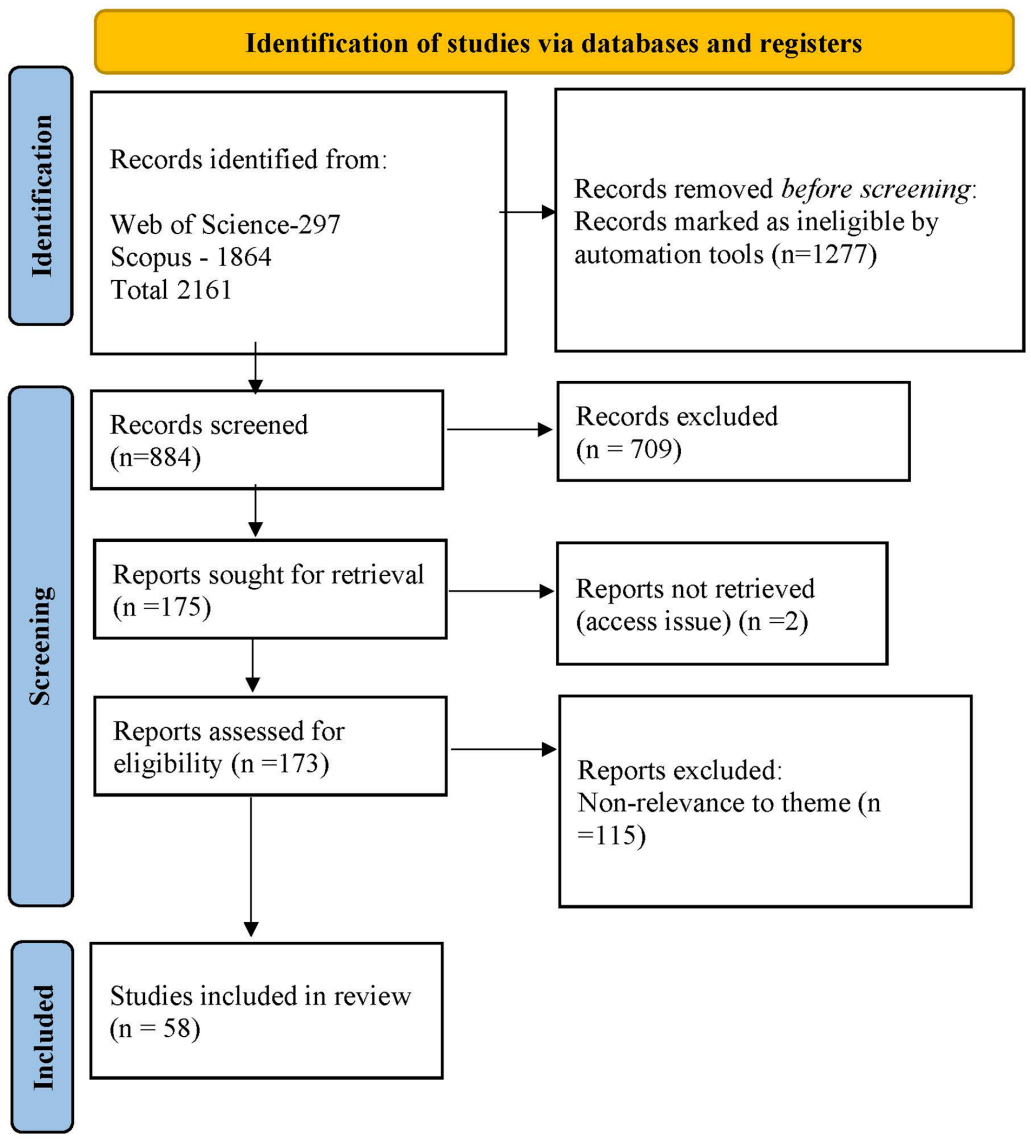
Flow diagram of article selection using PRISMA.

In the eligibility assessment stage, manual scrutiny of titles and abstracts led to the exclusion of 709 articles that diverged from the topic of gaslighting, i.e., gaslighting was neither the primary lens of the study nor the main finding. Additionally, access to two papers was limited to only abstract. After screening 173 articles, a total of 58 articles were identified as pertinent to understanding gaslighting phenomenon. Figure 1 presents the PRISMA flow diagram that delineates the article selection process. It is pertinent to mention that the conceptualization of gaslighting in various domains intertwines the behavior and experience of gaslighting while in the work context a scale has been developed to measure gaslighting behaviors (Kukreja and Pandey, 2023). However, beyond its behavioral operationalization, gaslighting represents a broader workplace phenomenon that integrates both behavioral and experiential dimensions and has yet to be fully theorized in a comprehensive and systematic way.

## Stage 2: identification of potential attributes of ‘gaslighting’

The objective of this phase of the definition process is to identify the potential attributes of gaslighting (Podsakoff et al., 2016). In this phase, we aggregate the definitions of the concept from research in fields other than organizational behavior, such as sociology, communication, politics, and other domains, based on papers selected in the systematic literature review. We then review the definition of gaslighting provided by scholars in organizational behavior. This enables us to delineate the definitions of the construct and lay out which attributes are necessary and which are contingent.

In Tables 1 and 2, we analyze the definition of gaslighting provided to identify the key attributes. After that, we categorize the attributes in individual definitions into necessary and contingent (Hillmann and Guenther, 2021; Podsakoff et al., 2016). For this, we carry out a literal interpretation of the definitions. For example, the use of the word ‘may’ before an attribute would be interpreted to refer to an optional attribute. However, the attributes that have no hedging words have been considered as the necessary attributes in that definition. Additionally, while doing this we bear in mind that there are two parties-the perpetrator (gaslighter) and the target (gaslightee). The gaslighting phenomenon is a combination of gaslighting behavior (action) by the perpetrator and the experience of gaslighting by the gaslightee (perception). Further, the experience of the gaslightee is dependent upon the power imbalance between the gaslightee and the gaslighter (Sweet, 2019) and not just the behavior of the gaslighter. Thus, gaslighting constitutes a complex phenomenon with two interrelated components: action, in the form of gaslighting behaviors, which is necessary, and perception, the experience of being gaslit, which is contingent.

**Table 1:** Definitions as given in other streams of scholarly research.

Area of research	Authors	Conceptualization of gaslighting	Key attributes
Sociology	Sweet (2019, p. 852, 855, 856)	“Set of attempts to create a surreal social environment by making the other in an intimate relationship seem or feel crazy.” “Specifically, gaslighting is effective when it is rooted in social inequalities, especially gender and sexuality, and executed in power-laden intimate relationships.” “The association of femininity with irrationality, which makes women more vulnerable to this form of abuse”	it is a behavior (necessary) to mislead or trick the target to distort target’s reality (necessary) target is in an intimate relationship with the perpetrator (necessary) power imbalance (necessary) self-doubt not obvious for all targets (contingent)
	Graves and Samp (2021, p. 3379)	“Gaslighting as a dysfunctional interpersonal dynamic, emerging from normative conflict patterns.” “Gaslighting is therefore a method of control that produces and reproduces dependence power.”	dysfunctional interpersonal dynamic (necessary) emerging from conflicts (necessary) power imbalance (necessary)
	Abu-Laban and Bakan (2022, p. 508, 510)	“Racial gaslighting has emerged as a key example of a broader pattern of what has been termed ‘structural gaslighting’, that is, a form of gaslighting designed by those with power to maintain the status quo over marginalised groups.” “Racial gaslighting operates through power inequities”	attempt to maintain status quo (necessary) power imbalance (necessary) trivialize—denial of experience of marginalized people (necessary) power imbalance (necessary)
Politics	Beerbohm and Davis (2023, p. 868, 869)	“We can draw together the shared properties from this conceptual work. At its core is the idea that its victims’ rational faculties have been captured in a distinctive way: an agent (A) wrongly induces another agent (B) to doubt B’s ability to respond rationally to evidence, in order to make B epistemically reliant on A.” “The significance of the identity of members of the target group finds support in sociological work on gaslighting. Sweet (2019, p. 852) explains that “gaslighting is effective when it is rooted in social inequalities, especially gender and sexuality, and executed in power-laden intimate relationships.”	attempting to induce doubt in the mind of the target as a rational individual (necessary) intention to make the target dependent on the perpetrator (necessary) power imbalance (necessary)
	Vernon (2024, p. 11)	“Gaslighting refers to a form of manipulation in which an abuser attempts to create self-doubt in the mind of the person they are attempting to victimize. This may be to convince them of their benevolence, or of the victim’s lack of worth, in order to perpetuate the cycle of abuse.”	form of manipulation (necessary) attempt to create self-doubt in target (necessary)
Communications	Graves and Spencer (2022, p. 1, 4)	“A dysfunctional communication dynamic in which one interlocutor attempts to destabilize another’s sense of reality” “We view gaslighting as a discursive dynamic drawing on reservoirs of social power to create language structures that destabilize a gaslightees sense of reality embedded in their knowledge claims.”	dysfunctional communication tactics (necessary) attempt to destabilize target’s sense of reality (necessary) power imbalance (necessary)
Psychology	Calef and Weinshel (1981, p. 52)	“It is, first of all, a piece of behavior in which one individual, with varying degrees of success, attempts to influence the judgment of a second individual by causing the latter to doubt the validity of his or her own judgment. The motivation may be conscious, although it is usually unconscious; and almost invariably the conscious motives are rationalizations and/or distortions of deeper, more complex, and less acceptable motives. The victim becomes uncertain and confused in regard to his or her assessment of internal or external perceptions and the integrity of his or her reality testing.”	it is a behavior (necessary) it can have conscious or unconscious motives (contingent) has varying degrees of success, i.e., self-doubt by target (contingent)
International Relations	Hansen (2023, p. 466)	In respect of post-colonial gaslighting: “identify the following levels to it: (a) withholding and refusing to engage in a conversation about certain things, (b) trivialising, belittling and disregarding someone’s feelings and beliefs, (c) diverting and removing focus and challenging the credibility of the person, (d) invoking stereotypes, particularly racialised and gendered, to delegitimise the victim’s account of reality and finally (e) denial or offering an alternative account, that directly contradicts the victim’s experience of reality” “…In this sense, using the concept of gaslighting as a heuristic to examine power dynamics is by no means novel.”	refusing conversation/discussion (necessary) trivializing feelings and beliefs (necessary) diverting/ removing focus from the target by challenging the credibility of the target (necessary) leveraging stereotypes (necessary) denial of claims of the target (necessary) offering contradictory account of situations/incidents (necessary) power-imbalance (necessary)
Environment	Jackson (2024, p. 1, 9)	“Climate resilience governmentality is a form of governmental gaslighting because it denies the lived experiences of precarity, insecurity and structural violence throughout regional Australia” “Resilience can be considered a form of governmentality, with certain rationalities, calculations and reflections that align with those who hold political and economic power.”	government as the perpetrator (necessary) trivialization through denial of lived experiences of precarity, insecurity and structural violence (necessary) power imbalance (necessary)
Philosophy	Abramson (2014, p. 2, 19)	“A form of emotional manipulation in which the gaslighter tries (consciously or not) to induce in someone the sense that her reactions, perceptions, memories and/or beliefs are not just mistaken, but utterly without grounds paradigmatically, so unfounded as to qualify as crazy.” “All of the gaslighting tools I discuss in what follows are especially efficient tools gaslighting insofar as they take place against a background of power inequities.”	form of manipulation (necessary) intention—implicit or explicit (contingent) attempt to distort target’s reality (necessary) power imbalance (necessary)
	Spear (2020, p. 230)	“Gaslighting involves (i) the attempt by the gaslighter to undermine his victim’s self-trust: her conception of herself as an autonomous locus of experience, thought, and judgment. The gasligther’s (ii) motivation is a strong desire to neutralize his victim’s ability to criticize him and to ensure her consent to his way of viewing things (specifically with regard to issues relevant to the relationship, perhaps in general), and thus to maintain control over her. The gaslighter (iii) pursues this goal by means of a strategy of manipulation, fabrication, and deception that (iv) specifically relies upon his victim’s trust in him as a peer or authority in some relevant sense.” “The motives of the gaslighter are consistent with “power and control” motives typically ascribed to abusers (Wagers, 2015).”	attempt to undermine the target (necessary) such that target doubts self (contingent) the perpetrator wants to control the target (necessary) the perpetrator may use tactics of manipulation, lying or may create false evidence (necessary) the gaslighter is trying to capitalize on the trust by the target on him as the knower (necessary) power imbalance (necessary)
	Stark (2019, p. 223, 224)	“Gaslighting occurs when a person (the “gaslighter”) manipulates another (the “target”) in order to make her suppress or doubt her justifiable judgments about facts or values. He does this by denying the credibility of those judgments using these two methods: First, the gaslighter sidesteps evidence that would expose his judgment as unjustified. Second, he claims that the target’s judgment lacks credibility because it is caused by a defect in her.” “Both epistemic and manipulative gaslighting require a power differential between the gaslighter and his target.”	manipulate target (necessary) attempt to make the target doubt self (necessary) deny evidence or credibility of target (necessary) power imbalance (necessary)
	Kirk-Giannini (2023, p. 757)	“For all persons A, B, and propositions p: A gaslights B with respect to p if (i) A intentionally communicates p to B, (ii) B knows (and A is in a position to know) that if p is true, then B has good reason to believe that she lacks basic epistemic competence in some domain D, (iii) A does not correctly and with knowledge-level doxastic justification believe p, and A does not correctly and with knowledge-level doxastic justification believe that B lacks basic epistemic competence in D, and (iv) B assigns significant weight to A’s testimony.”	intentional communication (necessary) communication of A’s belief that B is not competent in some domain (necessary) B holds A’s opinion valuable, i.e., power imbalance (necessary)
Medicine	Shapiro and Hayburn (2024, p. 34751)	“Medical gaslighting” as the psychological and institutional process that naturalizes the diminution of those who are testimonially disempowered within the context of the medical institution, and in their relationships with those who have authority to legitimize the patient’s illness experience.” This experience of medical gaslighting, we propose, is itself a mechanism for the creation of a particular form of medical trauma; through the experience of being gaslit in the context of their attempt to access medical assistance and treatment, the patient is traumatized. The particular form of trauma a patient will experience in such circumstances, is medical trauma.	is a process (necessary) trivializes the patient (necessary) power imbalance (necessary) the experience of being gaslit (necessary)
Technology	Cotter (2023, p. 1227)	“Black box gaslighting captures how platforms may leverage perceptions of their epistemic authority on their algorithms to undermine users’ confidence in what they know about algorithms and destabilize credible criticism.”	attempt to confuse users (necessary) use of power imbalance (necessary) causes harm and confidence loss to users (contingent)

**Table 2:** Definitions as given in work context.

Authors	Conceptualization of gaslighting	Key attributes
Jones (2023, p. 901)	“Gaslighting is posited as the deployment of tactics to make women doubt their sanity and as a means of securing personal advantage.” “Main features of gaslighting, such as the exercising of a power imbalance and victimization.”	deployment of tactics (necessary) attempts to make target doubt sanity (necessary) women are the target (necessary) attempt to gain personal advantage (necessary) power imbalance (necessary)
Catalano et al. (2024, p. 385)	“Administrative gaslighting describes how institutional dynamics support a false narrative that institutional change is possible through a one-time program or an individual’s efforts, and that an individual’s shortcomings are the reason for lack of advancement.”	false narrative by the organization (necessary) failure blamed on individuals rather than the policy/program (necessary)
Brewer (2021, p. 1083)	“The article uses the term institutional betrayal to describe gaslighting, where nurses who report issues in the workplace (i.e., whistleblowers) were made to feel that their complaints were insignificant or that issues in the workplace were not really happening. The resulting cognitive dissonance, which is internal confusion and doubt, among the person who is gaslighted creates a deep sense of emotional and psychological damage. Key attributes of institutional betrayal in nursing are (1) violating of trust or confidence by implicit or explicit means, (2) occurring between nurse and an institution to which the nurse belongs or identifies with (e.g., workplace, political institution, community, or society), (3) creating perceived constraints to ethical culture and practice, (4) occurring as covert and/or overt actions or behaviors, (5) creating or compounding stress, and (6) diminishing the sense of an ethical culture.”	trivializing of complaints at the workplace (necessary) directed towards whistleblowers (necessary) attempt to distort the target’s reality (necessary)

Table 1 highlights the definitions of ‘gaslighting’ as given by scholars in different fields of research. We treat self-doubt by the target as a contingent attribute of the gaslighting phenomenon since it is dependent on the power differential between the gaslighter and the gaslightee.

The conceptualization of gaslighting across various disciplines demonstrate common themes and unique attributes. Sociology defines gaslighting as a behavior aimed at creating a distorted reality, often in intimate relationships, making the target feel “crazy” (Sweet, 2019, p. 852). As per literature in politics, gaslighting has been conceptualized as an attempt to create epistemic doubt in the target to make the target dependent on the perpetrator (Beerbohm and Davis, 2023). Similarly, in communications research, gaslighting is viewed as a dysfunctional interaction that destabilizes the target’s sense of reality (Graves and Spencer, 2022). In philosophy, gaslighting is viewed as an emotional manipulation that leads the target to question the validity of their perceptions, memories, and beliefs, even if the perpetrator’s intent is implicit (Abramson, 2014). In psychology, gaslighting may be either conscious or unconscious and its success in distorting reality depends on the vulnerability of the target (Calef and Weinshel, 1981) and hence gaslighting is considered to be a tango between the gaslighter and the gaslightee (Stern, 2018). Gaslighting tactics in international relations take the form of discussion avoidance, downplaying of the target’s experiences, and invoking stereotypes to delegitimize the target’s reality (Hansen, 2023). In medicine, gaslighting is conceptualized as the tendency of doctors and nurses to trivialize patients’ symptoms based on systemic prejudices (Sebring, 2021). Gaslighting behaviors are more common in cases where individuals or institutions seek to maintain control or uphold the status quo, especially when dealing with marginalized groups (Vernon, 2024). Thus, based on these conceptualizations, gaslighting is a combination of manipulation, attempts to induce doubt, and the exploitation of power dynamics.

Table 2 presents definitions of gaslighting in the work context.

Jones (2023) describes gaslighting as a set of tactics aimed at women to make them doubt their sanity for the perpetrator’s gain. Catalano et al. (2024) conceptualizes administrative gaslighting, where institutions propagate false narratives about change, blaming individuals for systemic failures. Institutional betrayal is the lens used to trivialize whistleblower complaints to create confusion and emotional harm (Brewer, 2021). As seen in Tables 1, 2, gaslighting has been posited to be based on gender (Jones, 2023), race (Abu-Laban and Bakan, 2022), and intimate relationships (Sweet, 2019).

Before elaborating on the attributes of gaslighting, it is prudent to understand who can be a gaslighter and who can be a gaslightee. Gaslighting inherently involves two actors: a gaslighter and a gaslightee. Either role may be occupied by an individual or a collective entity. The perpetrator can be an organization (Brewer, 2021; Catalano et al., 2024), a government (Jackson, 2024), a political leader (Hansen, 2023), an individual (Jones, 2023), or a group (Abu-Laban and Bakan, 2022). Individuals who have higher power or status may use gaslighting tactics to assert and maintain control over their targets (Omran and Yousafzai, 2023; Sweet, 2019). It can be directed toward an individual (Sweet, 2019) or a group (Maeve Wall et al., 2023; Wood and Harris, 2024), and even citizens at large (Jackson, 2024). An organization itself, or groups within an organization, may perpetrate gaslighting against marginalized groups through misleading narratives or policies with systemic bias (Catalano et al., 2024; Jones, 2023; Storm and Muhr, 2023).

## Stage 3: thematic analysis of existing definitions

The repository of definitions was analyzed one by one above, and we assimilate these into themes to understand potential attributes that may be necessary and those that are contingent. The definitions were read and re-read to understand the common themes among these conceptualizations in scholarly literature.

### Theme 1: trivialization

Trivialization refers to undermining workers’ perspectives, fears, and realities, which is a common tactic in gaslighting behaviors (Kukreja and Pandey, 2023). This form of manipulation can manifest in various ways. For example, a supervisor may divert discussions to project faults onto the subordinate (Klein et al., 2023; Li and Samp, 2023; Omran and Yousafzai, 2023), tell them they are “imagining” things (Klein et al., 2023, p. 1327), or pass degrading comments followed by rewards to confuse the subordinate (Christensen and Evans-Murray, 2021). For instance, supervisors might be inconsistent between their words and actions, deny commitments, or undermine complaints and concerns raised by the subordinate (Kukreja and Pandey, 2023). Another aspect of trivialization involves supervisors twisting or misrepresenting the subordinate’s words or taking credit for their work (Abramson, 2014).

Trivialization aims to create doubt and insecurity in the target, causing them to question their experience, competence, judgment, or memory. In some cases, trivialization is used to avoid accountability (Klein et al., 2023). In line with various conceptualizations by scholars (e.g., Sebring, 2021; Graves and Spencer, 2022), this method of creating doubt by trivializing the experiences of the target is recognized as a fundamental attribute of the gaslighting behavior. By manipulating the target’s understanding of their own experiences, the perpetrator attempts to reinforce a distorted narrative that challenges the target’s perceptions and capabilities (Graves and Spencer, 2022). In some cases, perpetrators use trivialization tactics explicitly to discredit the target (Catalano et al., 2024; Christensen and Evans-Murray, 2021; Omran and Yousafzai, 2023). Thus, we posit that trivialization is a necessary attribute of gaslighting.

### Theme 2: affliction potential

It has been posited that affliction potential is a necessary attribute of gaslighting. Affliction refers to something that causes distress, pain, or suffering (Affliction, 2024). Within organizational contexts, gaslighting is inherently oriented toward gaining advantage or exerting control, with the potential to produce psychological, relational, reputational, or occupational harm to the target (Storm and Muhr, 2023).

Gaslighting may cause harm to the target’s personal well-being through sustained psychological manipulation. For example, a supervisor might engage in gaslighting behaviors toward a subordinate perceived as a threat, to undermine their confidence, hinder career progression or create dependency on the perpetrator (Abramson, 2014). Such behaviors may include exerting unnecessary control, encouraging the subordinates to become their own worst critics, or fostering dependency by making decisions on the subordinate’s behalf (Kukreja and Pandey, 2023), thereby contributing to psychological harm.

In addition, supervisors may engage in inconsistent interpersonal behavior, being supportive or kind at one moment and hostile or dismissive the next (Abramson, 2014; Kukreja and Pandey, 2023). They may also exclude subordinates from discussions or important meetings, thereby limiting access to critical resources and information (Abramson, 2014; Spear, 2020; Christensen and Evans-Murray, 2021). These practices can produce relational harm while simultaneously constituting occupational harm by obstructing performance, visibility, and advancement.

Further, affliction potential is not confined to downward hierarchical relationships. Occupational and reputational harm may also occur when a subordinate who feels threatened by a supervisor’s position engages in gaslighting tactics aimed at destabilizing or discrediting the supervisor (Graves and Spencer, 2022). In the workplace, such dynamics can result in occupational and reputational harm, where gaslighting may be driven by envy, jealousy, or a desire to neutralize a threat (Christensen and Evans-Murray, 2021). Additionally, organizational policies, such as work–life balance, designed to support female employees may be misused as gaslighting tools that delay career progression, further illustrating the potential for institutionalized occupational harm (Storm and Muhr, 2023). These examples indicate that gaslighting is characterized by an inherent potential to cause harm, whether harm is intentionally pursued or emerges as a consequence of efforts to gain advantage and control. On this basis, we argue that affliction potential constitutes a necessary attribute of gaslighting. The existence of harm potential is essential, although the manifestation of actual harm depends on the perceived power imbalance.

### Theme 3: intentionality

Intentionality in gaslighting can manifest either as a deliberate and explicit effort or as an unconscious and implicit attempt to influence or control others (Abramson, 2014). For example, in an organizational context, an employer, supervisor, co-worker, or subordinate might engage in behaviors, such as denying a promotion, neutralizing perceived threats, or securing a promotion for themselves through manipulative tactics. This suggests that while intentionality is considered inherent in gaslighting, it is not an attribute that must be demonstrated for the existence of gaslighting (Abramson, 2014; Field-Springer et al., 2022; Kukreja and Pandey, 2023). The possibility of unconscious manipulation implies that gaslighting can occur even without a deliberate intent to manipulate or destabilize another’s perception or status. In this regard, we also draw attention to the conceptualization of gaslighting as epistemic injustice (Omran and Yousafzai, 2023). Based on our conceptualization, workplace gaslighting constitutes epistemic injustice when it is grounded in identity-based prejudice that undermines the target’s credibility, and incidental injustice when such harm arises as a by-product of actors’ strategic efforts to advance or protect their interests. The identification and attribution of gaslighting behaviors do not require evidence of the gaslighter’s intent; rather, the focus remains on the nature of the behavior. The conceptualization treats gaslighting behaviors as empirically identifiable such that their classification does not depend on demonstrating the actor’s intentionality or assigning moral blame.

### Theme 4: self-doubt in the target

A perpetrator may employ manipulative tactics on the target to sow doubt in the target, but these attempts do not uniformly result in the target doubting themselves. This variability in the efficacy of gaslighting behaviors helps in understanding gaslighting phenomena such that the act of gaslighting and the state of being gaslit are different (Catapang Podosky, 2021). The experience of self-doubt for target due to exposure to gaslighting behaviors can vary based on their personal characteristics, the characteristics of the perpetrator and the specific context which can together impact power imbalance (Abramson, 2014; Dorri et al., 2024; Li and Samp, 2023; Sweet, 2019; Wood and Harris, 2024). Therefore, while the act of attempting to create doubt (gaslighting) is an action, the experience of the target doubting themselves (being gaslit) is a perception. This distinction is vital as it highlights that the behavior and experience of gaslighting are not synonymous; the action (to gaslight) and the perception (experience of being gaslit or not) are two separate parts of the entire gaslighting phenomenon.

### Dynamics of power in the workplace

Existing scholarship reveals that disparities in power and status are critical aspects of gaslighting behaviors (Graves and Spencer, 2022; Omran and Yousafzai, 2023; Sweet, 2019). Power in the workplace operates across multiple levels, filtering down from the societal level to the individual level via the organizational and interpersonal levels (Thatcher et al., 2023). Societal power imbalances are systemic in nature (Healy et al., 2011). At the societal level, power operates through broad structures such as norms, institutions, and labor markets, embedding meaning into identities and shaping opportunities and constraints that filter down into organizations (Thatcher et al., 2023).

At the organizational level, power is expressed through culture and climate, HR practices, and formal roles (Hwang and Beauregard, 2022; Storm and Muhr, 2023). Organizations may either reinforce or counteract biased power structures originating at the societal level. Organizations can implement equitable policies and empower managers to reduce the societal power structures.

At the interpersonal level, power is reflected in workplace relationships such as dyadic or team-based interactions, mentoring relationships, formal status positions, and informal networks. Individuals occupy positions and engage in workplace relationships in which power differentials exist. According to the foundational theories of power, six key sources are legitimate, reward, coercive, referent, expert, and informational (French and Raven, 1959; Raven, 2008). Recent literature places sources of power into structural position (formal or informal), demographics and morphology, competence, and personality (Anderson and Brion, 2014). Interpersonal power is rooted in perceptions individuals hold of others who are engaged in workplace relationships (Ragins and Sundstrom, 1989). Interpersonal power does not exist in isolation (Emerson, 1962); rather, it is embedded within and interacts with power at other levels.

Individual-level power stems from personal characteristics such as background, personal style, personality, and nonwork roles (Anderson and Brion, 2014; Ragins and Sundstrom, 1989). In addition, individuals can harness power derived from their individual differences or skills to influence their experience (Kipnis et al., 1980; Yukl and Falbe, 1990).

Thus, power may operate simultaneously and interact across societal, organizational, interpersonal, and individual levels, producing power imbalances that shape workplace relationships. Power is not solely hierarchy-based; it may also stem from informal, relational, and individual sources as explained above. These power imbalances influence the degree of trust and credibility afforded to the gaslighter by the gaslightee, thereby increasing the likelihood that the gaslightee will doubt their own judgments and rational capacities over those of the gaslighter. Accordingly, gaslighting in the workplace may arise from power imbalances rooted in both formal and informal sources, embedded within and reinforced by multilevel power structures.

## Stage 4: differentiating from related constructs

Workplace mistreatment includes abusive supervision, bullying, discrimination, exclusion/ostracism, general harassment, incivility, interpersonal conflict, psychological aggression, sexual harassment, undermining, and violence (Dhanani et al., 2021). Accordingly, we differentiate gaslighting behaviors from each of these negative workplace behaviors. In addition, because gaslighting is frequently conflated with disagreement, we also distinguish it from disagreement to further clarify its conceptual boundaries.

### Abusive supervision

Abusive supervision comprises continuous display of verbal and nonverbal hostile behaviors, excluding physical contact (Tepper, 2000). In contrast, a gaslighter may not necessarily display hostility; rather, they may employ charm to confuse and distort the target’s perception of reality (Stern, 2018). Thus, while a gaslighter may sometimes manifest hostility, it is not a requisite characteristic of gaslighting. For example, if the perpetrator makes offensive remarks towards the target (e.g., ideas are stupid), they might then claim it was merely friendly banter or just a joke (Catapang Podosky, 2021) in case of gaslighting. However, it may become abusive supervision if the perpetrator stands by their inappropriate remarks (Tepper, 2000).

### Bullying

Einarsen (2000) describes bullying as “situations in which individuals are subjected to continuous negative acts, such as “abuse, offensive remarks, teasing, ridicule, or social exclusion,” by co-workers, supervisors, or subordinates over an extended period. While there are similarities between certain behaviors in gaslighting and bullying, gaslighting distinctively involves the perpetrator minimizing the target’s experiences in an effort to make them question their own perceptions of these acts, while no such denial or attempts are made in the case of bullying (Kukreja and Pandey, 2023). The tactics used in bullying are direct whereas gaslighting involves covert and subtle tactics (Jones, 2023). Further, gaslighting does not necessarily involve overt aggression while bullying involves aggression (Einarsen, 2000). Bullying is usually confrontational whereas in gaslighting the perpetrator maintains appearances or appears concerned for the victim (Jones, 2023). However, due to the overlap of characteristics of bullying with gaslighting, scholars have also termed gaslighting as a “pernicious form of bullying” (Christensen and Evans-Murray, 2021, p. 1).

### Discrimination

Workplace discrimination refers to the disadvantage faced by individuals belonging to a certain ‘social category’ compared to other groups with similar potential or demonstrated success (Dipboye and Halverson, 2004). Unlike workplace discrimination, which is contingent on social category membership, gaslighting is not always identity dependent and can be enacted at the individual level as a form of psychological manipulation (Abramson, 2014).

### Exclusion/ostracism

Ferris et al. (2008) describe exclusion or ostracism as the feeling of being ignored or left out. In gaslighting scenarios, exclusion may be used strategically and intermittently by framing it as an accidental mistake. This exclusion is not comprehensive; the target may remain included in other activities. By alternating between exclusion and inclusion, the perpetrator maintains plausible deniability while destabilizing the target through inconsistent behavior.

### Incivility

Workplace incivility is a subtle, low-intensity deviant behavior that ambiguously intends to harm the target, contravening standards of mutual respect (Andersson and Pearson, 1999). Incivility and gaslighting are both subtle in nature but a gaslighter might use manipulative tactics while remaining respectful (Storm and Muhr, 2023). Hence, displaying disrespect is not a necessary condition for gaslighting behavior.

### Psychological aggression

Schat and Frone (2011) describe psychological aggression as behaviors characterized by verbal or symbolic acts that usually result in immediate psychological harm. Conversely, gaslighting may cause psychological harm, usually more gradually, unfolding over a longer span (Abramson, 2014).

### Sexual harassment

Sexual harassment refers to unsolicited sexual advances, requests for sexual favors, and other forms of verbal or physical harassment of a sexual nature (The Equal Employment Opportunity Commission, 2015). On the contrary, gaslighting may be gendered but it is usually not sexual in nature.

### Undermining

Undermining hinders an employee’s capacity to cultivate positive interpersonal relationships, succeed at work, and maintain a favorable reputation (Duffy et al., 2006). A gaslighter uses tactics to gain control over the target for personal advantage (Spear, 2020). Undermining is closer to the attribute of trivialization of gaslighting but undermining does not centrally emphasize perception destabilization, which is the core of gaslighting.

Gaslighting may have some commonality with abusive supervision, bullying, discrimination, ostracism, and sexual harassment but has attributes that are different from these, as explained above. However, it is relevant to note that these forms of mistreatment are not mutually exclusive. An employee who experiences sexual harassment, bullying, or discrimination may also be subjected to gaslighting. For example, when the perpetrator subsequently denies the misconduct, trivializes the target’s experience, or attempts to make the target doubt their interpretation of events (Abramson, 2014; Maeve Wall et al., 2023; Sweet, 2019; Wood and Harris, 2024). Thus, gaslighting can co-occur with, reinforce, or compound other forms of workplace mistreatment.

**Figure 2:**
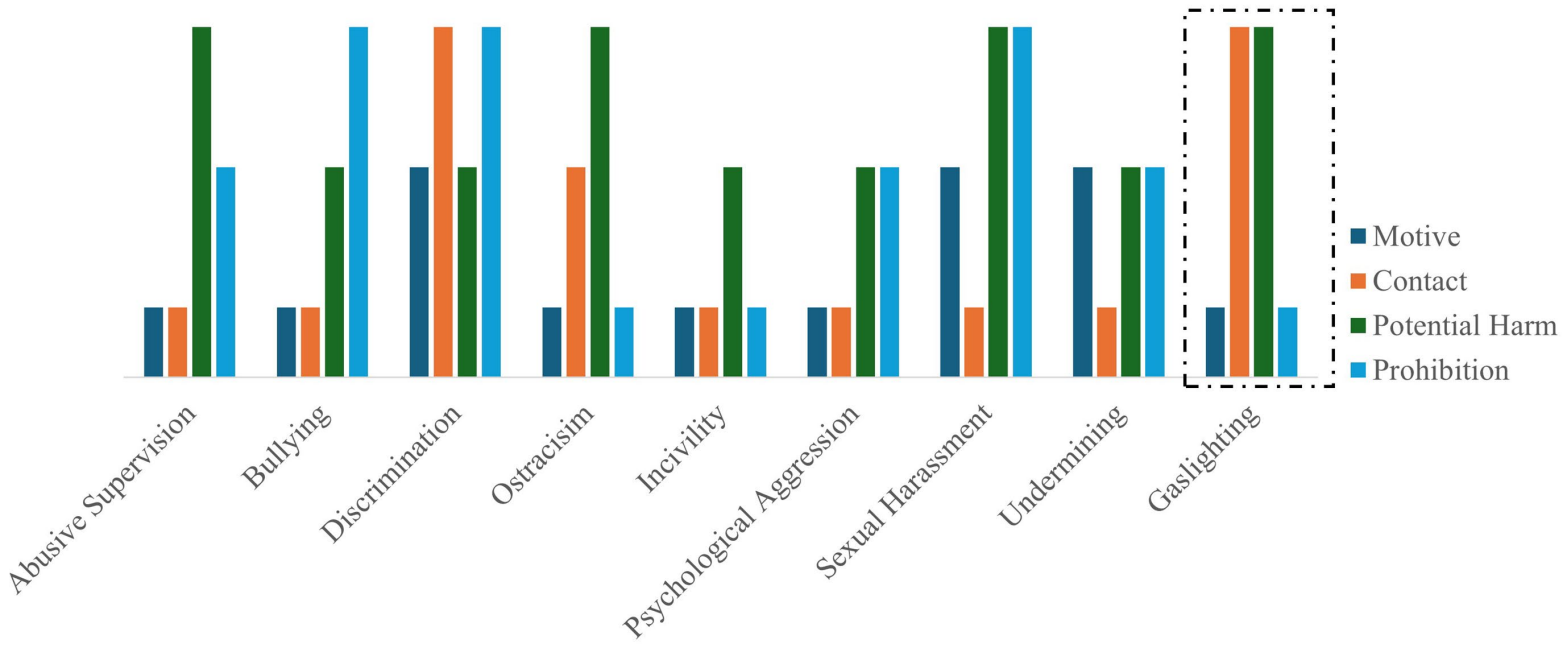
Comparison across workplace mistreatment constructs.

Further, a recent paper on workplace mistreatment suggests that mistreatments can be classified using the taxonomy of motive, contact, harm, and prohibition (Dhanani and Bogart, 2025). Gaslighting is characterized by an unspecified or ambiguous motive, the use of both acts of commission and omission, and the potential to affect the target’s psychological state. Due to its subtle and covert nature, it is not explicitly prohibited by law. Further, unlike other forms of mistreatment, gaslighting uniquely targets epistemic and interpretive resources (Abramson, 2014; Catapang Podosky, 2021; Kukreja and Pandey, 2023; Spear, 2020). Based on the four dimensions proposed by Dhanani and Bogart (2025), we developed a graphical representation to visually map various workplace mistreatment constructs (refer Figure 2). However, we disagree on the aspect that sexual harassment causes less harm than other mistreatment constructs. We coded it as having a higher potential for harm than other constructs. The purpose of this visualization is to demonstrate that gaslighting represents a distinct configuration formed by the combination of all four dimensions. Because these dimensions are categorical rather than scale variables, the height of the graphical elements does not indicate magnitude or value; instead, it reflects categorical positioning only.

For visualization purposes, each dimension was coded as follows: Motive (non-specific = 1; specific = 2), Contact (commission = 1; omission = 2; both = 3), Potential Harm (less = 1; average = 2; more = 3), and Prohibition (not prohibited = 1; variable = 2; prohibited = 3).

### Disagreement

In addition to the above, it is important to distinguish disagreement from gaslighting even though it is not a workplace mistreatment. “A disagreement between A and B (where A and B are either individuals or groups of individuals) is a case where A accepts P and B rejects P” (Potter, 2013, p. 23). Expressions of disagreement, critique, or concern by a supervisor, co-worker, or subordinate, when grounded in legitimate reasons, do not constitute gaslighting. For example, when a supervisor provides evidence-based feedback indicating that an employee’s performance falls short of established expectations or requirements, such feedback should not be classified as gaslighting. Rather, gaslighting arises when concerns or contributions are trivialized, dismissed, or invalidated without explanation or factual basis, and when the individual’s competence, memory, or perception of reality is directly called into question.

## Stage 5: development of the conceptual definition

Having completed the thematic analysis and distinguished workplace gaslighting from other forms of workplace mistreatment, this section deals with its conceptualization. Workplace gaslighting phenomenon comprises gaslighting behaviors by gaslighters (necessary) and experience of being gaslit by gaslightees (contingent).

Workplace gaslighting behaviors (actions) are manipulative actions embedded in work relationships towards one or more persons or groups. These behaviors may be intentional or unintentional but involve trivializing work or concerns (necessary) and have affliction potential (necessary) for the target. These behaviors may be used to exert control, gain an advantage over others (individuals and/or groups of employees), or avoid accountability and criticism.

Gaslighting behaviors (action) may or may not impact the gaslightee; rather, the experience of gaslighting (perception) by the target is dependent on the perceived power imbalance. Hence, being gaslit is a contingent attribute of the gaslighting phenomenon. In severe cases of power imbalance, the target may experience self-doubt, confusion, or distortion of reality in terms of memory, judgement, or competence such that it undermines their ability to assert themselves, respond effectively, or maintain their position in the workplace.

Workplace gaslighting behaviors can manifest in various ways, including but not limited to:

- Labelling someone as irrational, oversensitive, or “crazy” (Abramson, 2014; Omran and Yousafzai, 2023; Sweet, 2019)
- Twisting facts, lying, or reframing events to suit the manipulator’s narrative (Gass and Nichols, 1988; Omran and Yousafzai, 2023; Spear, 2020)
- Refusing to address issues or engage in meaningful discussions (Graves and Samp, 2021; Li and Samp, 2023)
- Making promises or verbal commitments and later denying them (Li and Samp, 2023; Omran and Yousafzai, 2023)
- Giving vague or evasive responses to avoid clarity, resolution, accountability or criticism (Christensen and Evans-Murray, 2021; Klein et al., 2023; Tobias and Joseph, 2020)
- Excluding individuals from important communications, meetings, or decisions (Abramson, 2014)
- Taking credit for someone else’s work or achievements (Abramson, 2014; Christensen and Evans-Murray, 2021)
- Implementing policies, such as anti-discrimination or DEI initiatives, that exist only for appearances but lack genuine enactment or enforcement (Catalano et al., 2024; Storm and Muhr, 2023)
- Discrediting the victim as a rational person (Beerbohm and Davis, 2023; Stark, 2019)

**Figure 3:**
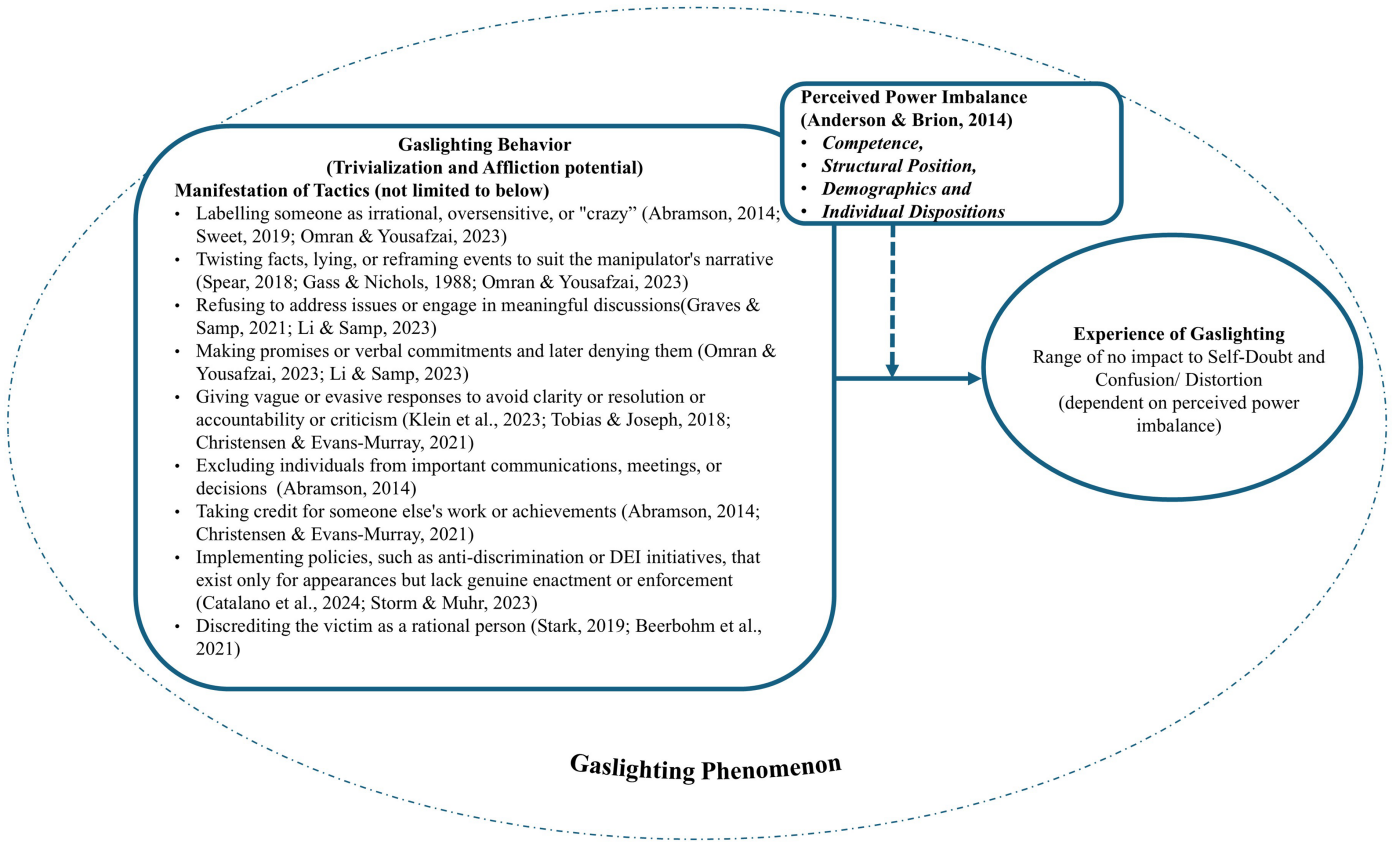
Conceptualization of workplace gaslighting phenomenon.

Based on the literature, we propose the conceptualization of workplace gaslighting phenomenon in Figure 3.

This conceptualization of the gaslighting phenomenon into action and perception is in contrast with other workplace mistreatment constructs. For example, in case of bullying, incivility, abusive supervision etc. the manifestation of the mistreatment is the negative act itself. However, in case of gaslighting, the experience of gaslighting is different than the act of gaslighting. The action comprises manipulative tactics while the experience is in the form of self-doubt, confusion and distortion of reality. This implies that gaslighting behaviors (trivialization & affliction potential) are therefore the necessary part of the phenomenon but the experience of being gaslit is contingent.

## Theoretical underpinnings of gaslighting in other domains

### Psychology perspective

In the psychology domain, the most frequently cited work on gaslighting is Stern (2018) (originally published in 2007) self-help book, *The Gaslight Effect*. It examined the roles of both the gaslighter and the gaslightee in the phenomenon. The gaslighting encounter was referred to as a ‘tango’, necessitating the involvement of both sides. The targets show commitment towards relationships and fear the failure of the relationship which is exploited by the gaslighters to compel targets into rationalizing and submitting to the actions of the gaslighters. Research under this perspective has looked into the personality factors of gaslighters and gaslightees. Individuals with neuroticism, intolerance of uncertainty, and heightened sensory processing have been associated with greater susceptibility to being gaslit (Hightower, 2017). Another study revealed that both gaslighters and gaslightee exhibited psychoticism and disinhibition (Miano et al., 2021). High levels of sadism and machiavellianism have been found to be related to the acceptability of gaslighting (March et al., 2025).

### Sociological perspective

The sociological theory of gaslighting conceptualizes gaslighting as a social phenomenon that exploits power imbalance in relationships to create an environment of “surreality” that undermines the target (Sweet, 2019). This view explains that the effectiveness of gaslighting tactics stems from the perpetrator’s ability to exploit systemic inequalities and leverage power imbalances rooted in gender, race, and class. The sociological perspective has also been extended in the medical context to study experiences of marginalized individuals (Li and Samp, 2023; Sebring, 2021). Unlike the psychological perspective that views gaslighting as a tango, this perspective posits that gaslighting is not a dynamic of mutual participation, but a powerful strategy of control made effective by pre-existing societal and institutional power structures.

### Social psychology perspective

A recent paper has combined the social and psychological perspective and offered a framework based on Prediction Error Minimization (PEM) theory (Klein et al., 2025). The core assumption is that close relationships inherently fulfil important epistemic needs, i.e., individuals depend on close others to shape and verify their self-views and their understanding of the world. This reliance fosters epistemic trust in the close other, which then provides the gaslighter with the necessary epistemic leverage to manipulate the target’s perception of reality. Within the PEM framework, gaslighting unfolds as a learning process through which the target comes to perceive themselves as epistemically incompetent due to the gaslighter’s consistent actions. The manipulation unfolds as the gaslighter generates salient prediction errors (e.g., by creating inconsistencies or altering the environment) and then offers explanations that subtly or explicitly suggest that the target is making errors. Rather than challenging the gaslighter’s trustworthiness, a prior belief that is robustly maintained in close relationships, the target is biased to accept the gaslighter’s explanations (a process called perceptual inference), thereby updating their internal model to reflect their own supposed incompetence. This repeated cycle of manufactured prediction errors and manipulative explanations gradually erodes the target’s agency and their faith in their own epistemic abilities, leading to increased dependence on the gaslighter as an authority having credibility.

## Discussion

Current research posits gaslighting as an intertwined phenomenon combining the actions of the gaslighter and the resulting impact on the gaslightee (Abu-Laban and Bakan, 2022; Jackson, 2024; Stark, 2019). Scholars have also described it as a form of dysfunctional communication (Graves and Spencer, 2022) that often emerges from conflict (Graves and Samp, 2021). In contrast, this paper conceptualizes the gaslighting phenomenon as having two distinct parts. First, the action which is the manipulative behavior of the gaslighter and second, the perception, i.e., how the gaslightee perceives the actions of the gaslighter. This perception can be on a spectrum of no impact to a severe impact of self-doubt. Such a distinction helps in identifying and resisting the maladaptive behavior of gaslighting, independent of perceived impact. Power imbalance shapes the perception of the manipulative behavior of the gaslighter by the gaslightee such that similar actions can have different responses (Abramson, 2014; Dorri et al., 2024; Li and Samp, 2023; Sweet, 2019; Wood and Harris, 2024). Further, we conceptualize power as a multilevel structure embedded within societal, organizational, interpersonal, and individual domains that conditions the credibility attributed to the gaslighter and, in turn, determines whether manipulative behaviors are internalized, resisted, intensified, or mitigated by the gaslightee.

## Recommendations

Based on the understanding of workplace gaslighting phenomenon, human resource managers can implement training programs to increase employee awareness, enabling timely identification and intervention (Leake et al., 2025). Further, organizations can develop policies to reduce workplace gaslighting.

## Future research directions

While this paper is conceptual in nature to explore the contours of workplace gaslighting, we encourage future researchers to build on this conceptual foundation to empirically test this multidimensional construct. The dimensions of power dynamics in workplace settings remain underexplored, presenting opportunities for future research to advance understanding of power sources beyond those rooted in social identity and their potential influence on gaslighting behaviors at work. Future research may also look into mechanisms through which different bases of power within workgroups are misused not only by supervisors, but also by peers and subordinates to enact gaslighting tactics. Qualitative research designs could be valuable in examining how power imbalances shape the perceptions and experiences of gaslightees. Given the potentially escalating nature of gaslighting, future research may study the process of being gaslit. Given the manipulative nature of gaslighting and the possibility that such behavior may be unintentional, empirical investigations are often limited to the perspective of the gaslightee. Future research could develop innovative methodological approaches to capture broader perspectives, including identifying which gaslighting behaviors are perceived as acceptable by employees and examining the individual characteristics and situational conditions under which gaslighting is more likely to occur. Additionally, longitudinal survey studies could be employed to assess the work-related and non-work-related outcomes affected by workplace gaslighting over time.

## Conclusion

Workplace gaslighting is a covert form of mistreatment that is inherently difficult to recognize. By distinguishing between manipulative action (necessary) and perceptual internalization (contingent), and embedding the phenomenon within multilevel power dynamics, this paper clarifies its defining attributes and differentiates workplace gaslighting from related constructs. We position workplace gaslighting as a distinct configuration of trivialization, affliction potential, and power-conditioned perception. This conceptual piece advances scholarly understanding and stimulates further research in this domain.
